# Measuring Spatiotemporal Parameters on Treadmill Walking Using Wearable Inertial System

**DOI:** 10.3390/s21134441

**Published:** 2021-06-29

**Authors:** Sofia Scataglini, Stijn Verwulgen, Eddy Roosens, Robby Haelterman, Damien Van Tiggelen

**Affiliations:** 1 Center for Physical Medicine and Rehabilitation, Military Hospital Queen Astrid, Rue Bruyn 200, 1120 Bruxelles, Belgium; eddy.roosens@mil.be (E.R.); damien.vantiggelen@mil.be (D.V.T.); 2 Department of Mathematics, Royal Military Academy, Rue Hobbema 8, 1000 Bruxelles, Belgium; rob.haelterman@mil.be; 3 Department of Product Development, Faculty of Design Science, University of Antwerp, 2000 Antwerp, Belgium; stijn.verwulgen@uantwerpen.be

**Keywords:** spatiotemporal parameters, treadmill, gait analysis, gait measuring system, wearable sensors

## Abstract

This study aims to measure and compare spatiotemporal gait parameters in nineteen subjects using a full wearable inertial mocap system Xsens (MVN Awinda, Netherlands) and a photoelectronic system one-meter OptoGait^TM^ (Microgait, Italy) on a treadmill imposing a walking speed of 5 km/h. A total of eleven steps were considered for each subject constituting a dataset of 209 samples from which spatiotemporal parameters (SPT) were calculated. The step length measurement was determined using two methods. The first one considers the calculation of step length based on the inverted pendulum model, while the second considers an anthropometric approach that correlates the stature with an anthropometric coefficient. Although the absolute agreement and consistency were found for the calculation of the stance phase, cadence and gait cycle, from our study, differences in SPT were found between the two systems. Mean square error (MSE) calculation of their speed (m/s) with respect to the imposed speed on a treadmill reveals a smaller error (MSE = 0.0008) using the OptoGait^TM^. Overall, our results indicate that the accurate detection of heel strike and toe-off have an influence on phases and sub-phases for the entire acquisition. Future study in this domain should investigate how to design and integrate better products and algorithms aiming to solve the problematic issues already identified in this study without limiting the user’s need and performance in a different environment.

## 1. Introduction

Gait is the main activity of human beings and corresponds to their physiological path of movement [1]. Physiological walking is a bipedal and complex activity. It corresponds to an automated, neuro-muscular activity that voluntary control can modulate [2]. Monitoring spatiotemporal parameters of gait (SPT) is important to assess performance [3] and abnormalities [4,5,6,7] and predict overuse injuries [8]. 

Gait is characterized by the walking cycle that is a representation of the succession of body movements. This succession of movement lasts a certain time comprising successive gait cycles. One gait cycle (100% time) is divided in two phases: the stance phase (60% of the cycle) and the swing phase (40% of the cycle) [9,10]. These two phases are additionally divided into several sub-phases. The stance phase (60% of the gait cycle) is successively divided into four sub-phases: the loading response (0–10%), the mid-stance (10–30%), the terminal stance (30–50%) and the pre-swing (50–60%) [9,10]. Meanwhile, the swing phase (40% of the gait cycle) is subdivided into three sub-phases: initial swing (60–73%), mid-swing (73–87%) and terminal swing (87–100%) [9,10]. During the gait cycle, spatial (or distance parameters) and temporal parameters can be measured with different measurements systems. Spatial parameters are the step length (right heel to left heel), step width (medio-lateral distance between two heel strikes) and the stride length (right heel to right heel), while the temporal parameters are the cadence (number of step per minute), speed, step and stride time, time and duration of phases and sub-phases [9,10].

Several measurements systems are used for monitoring spatiotemporal parameters in gait analysis [11], such as force or pressure plates [12], optoelectronic systems [13,14], wearable motion capturing systems (e.g., accelerometers [15], gyroscopes, magnetometers or the fusion of the three in an inertial measurement unit (IMU) [16,17,18], wearable pressure insoles [19] and smart clothes [20,21,22]).

Among these systems, IMUs were proved to be useful to monitor gait kinematics in an ecological approach not necessarily confined to the lab [8,15,17,23,24,25]. In addition, photoelectric cell systems such as OptoGait^TM^ composed of transmitting and receiving LED-bars are already used as transportable systems for monitoring spatiotemporal parameters indoor and outdoor [14,26,27]. Each bar can be connected to constitute a walkway with transmitting and receiving bars of several meters for monitoring gait overground, or on a treadmill.

There are differences between walking on the treadmill and overground. Treadmill walking offers a fixed environment, unlike walking on the floor. The contradictory effect between the visual field (the absence of retinal slippage) and the lower limbs affects walking speed and requires compensation which will result in an increase in the support phase to ensure its stability [28].

In most treadmills, substantially less energy is exerted compared to overground walking at the same speed, as smaller horizontal forces are exerted. As energy transfer is different, gait/walking characteristics are likely to be affected, especially in the propulsion phase [28].

Although instrumented gait analysis (IGA) systems can measure SPT in a different manner with a different sample frequency, it is also important for evaluating the accuracy and the method used to identify the heel strike and the toe-off events that can influence the calculation of the phases and sub-phases and consequently the step length and the cadence.

Rudish et al. [29] already evaluated the absolute agreement and consistency between five different IGA systems (two inertial, two pressure sensors and one optical). They found high agreement and consistency in gait cycle time, cadence, gait speed and stride length variables. However, poor agreement in determining phase and sub-phases caused by an inaccurate detection of toe-off and heel strike was found. Kluge et al. [30] studied the validity and test-reliability of an inertial system composed of two Shimmer3 sensors (Shimmer, Dublin, Ireland, 102.4 Hz) for the assessment of SPT using an optical markerless motion capture system (Simi Reality Motion Systems, Unterschleißheim, Germany, 100 Hz). They found a good test reliability for all parameters (intraclass correlation (ICC) > 0.81) except for gait velocity (ICC > 0.55). At the same time, Washabaugh et al. [31] studied the validity and repeatability of IMUs (APDM’s Mobility Lab) for SPT in overground and in treadmill using two different body placements (ankle and foot) demonstrating more repeatability when the sensors are placed on the feet. 

In addition, Lee et al. [32] evaluated the validity and reliability of the OptoGait^TM^ system for the assessment of SPT using the GaitRite system as a reference for the SPT measurements and found a correlation with all the SPT by ICC (3, 1) = (0.785–0.952), coefficients of variation (CVME = 1.66–4.06%), 95% limits of agreement, standard error of measurement (SEM = 2.17–5.96%) and minimum detectable change (MDC95% = 6.01–16.52%). However, no previous study was found that evaluate the agreement between OptoGait^TM^ and the inertial system Xsens (MVN awinda, Netherlands) on treadmill walking in healthy males. The study of the validity and reliability of IGA instruments is an essential asset that must be considered in clinical practice. This especially in the case of a new IGA instrument such as a wearable inertial mocap system that is not considered the gold standard as an optical system [16,33]. In fact, according to Muro-de-la-Herran [11] and Najafi et al. [34], the difference of using wearable respects not wearable sensors as optical systems is the possibility of evaluating the subject in a non-confined lab on different terrain and distances promoting patient’s autonomy and active role enhancing usability. However, these systems that are created to track movements are sometimes not created for clinical use as for assessing spatiotemporal parameters in gait analysis such as Xsens. According to Routhier et al. [35], a better study of these devices can allow more acceptance by the end-users that are also clinicians. Moreover, study these technologies is also important for mathematicians, designers and engineers. Design and technical issues can also affect the accuracy of these technologies [33,36,37,38].

Starting from these observations this study aims to measure and compare spatiotemporal gait parameters in nineteen subjects using a full wearable mocap system Xsens and a photoelectronic systems 1 m OptoGait^TM^ (Microgait, Italy) on treadmill walking imposing a walking speed of 5 km/h. 

## 2. Methods

Nineteen healthy male volunteers participated in the study signing informed consent. This study was also approved by an ethical committee (CE2019/32). Anthropometric characteristics of the population were collected before each subject’s acquisition, as shown in Table 1. 

**Figure 1:**
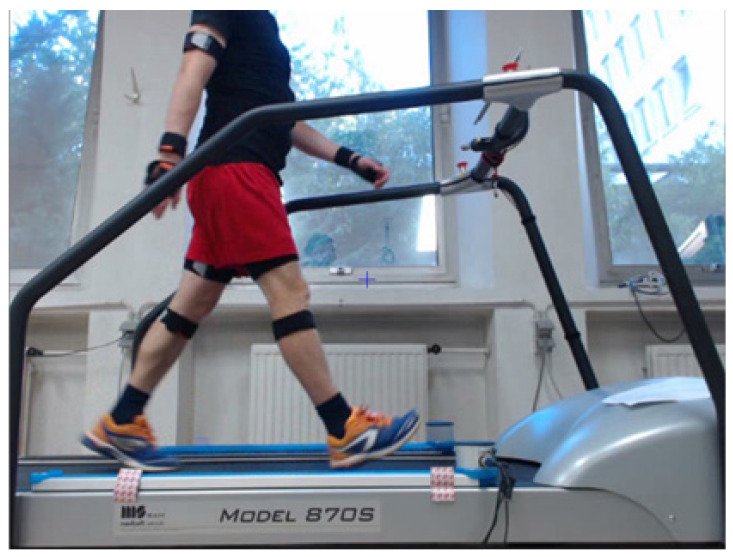
Acquisition of the subject wearing the Xsens (MVN awinda, Netherlands) full mocap system while walking between the optical bars, Optogait^TM^ (Microgait, Italy) on the treadmill (Medisoft, Model 870S, Belgium) at an imposed speed of 5 km/h.

The nineteen subjects wore the full mocap system walking at an imposed speed of 5 km/h on the treadmill (Medisoft, Model 870S, Belgium) with two photoelectronic optical bars (1 m) OptoGait^TM^ placed at both sides, Figure 1. The initial contact of the right foot was used as the reference point for the start of the experiment. The spatiotemporal parameters are the speed (m/s), the step length (m) and the phases of the gait cycle (s). 

### 2.1. Instrumentation

#### 2.1.1. The Optogait^TM^

The Optogait^TM^ system (1000 Hz) is a photoelectronic system consisting of two LED bars (96 light-emitting or light-receiving diodes) of one meter each. The device identifies interruptions in communication between the bars caused by the patient’s movement once it is positioned on the treadmill and estimates the duration and position. Spatiotemporal parameters are calculated automatically using a dedicated software provided by Microgait (Bolzano, Italy). 

#### 2.1.2. The Xsens

Xsens (MVN Awinda, Netherlands, 60 Hz) is a wearable inertial full mocap system composed of 17 inertial measurement unit Xsens attached to the body by band or attached to the upper body on a shirt. Kinematic data (3D position, linear and angular acceleration and velocity of twenty-three segments; the 3D joint angles of twenty-two joints, the centre of mass of the body, the 3D orientation, free accelerations and magnetic data of the seventeen wearable inertial systems) are captured and successively visualized by using the MVN Analyse software. Moreover, the software is not able to calculate automatically all the spatiotemporal parameters such as cadence, phases or step length.

1. The Inverted Pendulum Model

In this case all the spatiotemporal parameters were described as a percentage of the gait cycle, modelled as an inverted pendulum [20,21,39,40]. This inverted pendulum model [20,21,39,40] can be written in the form of an equation which describes the swinging movement of the legs according to:

(1) $${\mathrm{SL}}_{1}=2\sqrt{\left(2*L*h\right)-h^{2}}$$

where SL_1_ is the step length (m), L is the length of the leg (m) expressed as “the summit between the iliac crest and the floor” [41] and h is the amplitude of the center of mass (COM) oscillation during a walking pattern [20,21,39,40]. 

Using Equation (1) it is possible to determine the speed S_1_ (m/s) as:

S_1_ = (SL_1_/60) ∗ f ∗ α (2)

where f represents the cadence (number of step per minute) and α = 1.14 is a constant determined by the calibration considering the imposed speed [42].

2. Identification of Gait Phases and Sub-phases

**Figure 2:**
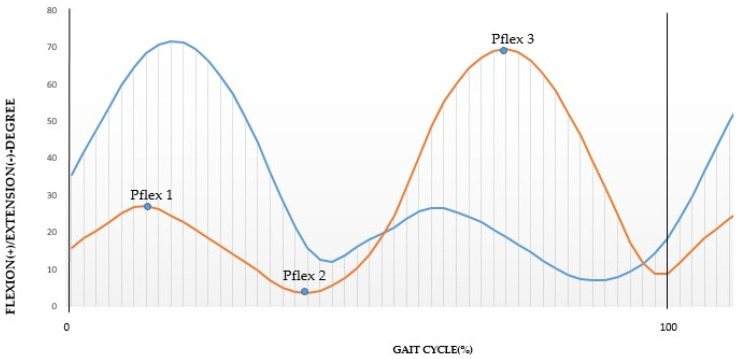
Key points on the knee angle wave in the sagittal plane during normalized walking.

The variation in the angle of the knee in flexion-extension in the sagittal plane (Figure 2) can be used to determine the different parts of the walking cycle [5,10]. This method is limited to the analysis of movements in the sagittal plane, but walking takes place in several planes [18].

Considering in this graph three specific peaks according to Abid et al. [5], it is possible to determine a stance phase peak angle (Pflex1), a swing phase peak flexion angle (Pflex2) and a local maximum (Pflex3), Figure 2. These key points are fundamental to determine the principal phases.

**Figure 3:**
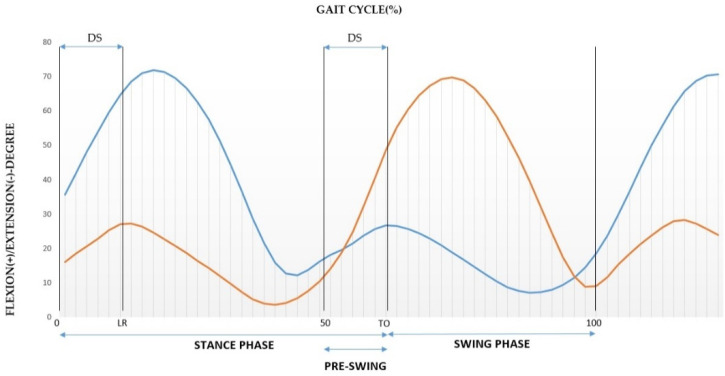
Representation of the knee joint angle and determination of the gait phases and sub-phases for a full stride with the right leg (which here represents the ipsilateral leg in blue) and the left leg (which here represents the contralateral leg in orange).

In addition to determine the sub-phases it is possible to consider the waves representing the flexion and the extension of the bilateral knees during a stride as it was shown in Figure 3.

The first flexion wave indicates the absorption phase of the body weight on the supporting leg, corresponding in the stance phase to the loading response (LR) during the first double support phase (DS) Figure 3 [5,20,21].

Then, as the opposite leg begins its cycle at 50% offset from the reference leg it is possible to determine the DS of the supporting leg and DS of the contralateral leg that corresponds to the pre-swing phase with toe-off of the supporting leg, Figure 3. Next is the swing phase composed by initial, mid and terminal swing constituting the 40% of the gait cycle (Figure 3).

#### 2.1.3. The Anthropometric Approach 

The anthropometric approach is a method that does not consider any IGA instrument but the anthropometric characteristics [43]. This method that is normally used in pedometers does not consider the COM and the step length variation. In this case, the step length SL_2_ (m) is every time fixed and is expressed as follow:

SL_2_ = C ∗ R ∗ 0.01 (3)

where R is the stature (m) and C is an anthropometric coefficient (C=0.415) representing the male population [43].

Considering the Equation (3) is possible to determine the speed (m/s) as follow:

S_2_ = ((SL_2_ ∗ ɣ)/60) (4)

where f represents the cadence and ɣ = 0.905 is a constant determined by the calibration with respect to the imposed speed on a treadmill at 5 km/h [42].

### 2.2. Statistical Analysis

Mean standard deviation (SD) of the twelve SPT variables (stance phase (s) and (%), swing phase (s) and (%), gait cycle (s), double support (s) and loading response (s) and (%), pre-swing (s) and (%), cadence (step/min), speed (m/s)) measured using the two IGA systems (Xsens, Optogait^TM^) were performed using the software Microsoft Excel (2019). Bland–Altman analysis was applied for understanding the agreement between these SPT variables using the two IGA systems together with the mean of differences with the 95% confidence intervals and *p*-values [44,45,46,47,48]. In addition, intraclass correlation coefficients (ICC) for mean of different gait variables looking for the absolute agreement and consistency and repeatability were studied using the software SPSS (IBM SPSS Statistics Version 27) [49,50]. Additionally, mean square errors (MSE) of the speed measured using the two systems (Xsens, Optogait^TM^) with respect to the imposed speed (5 km/h) of the treadmill were calculated [51,52,53].

## 3. Results

**Figure 4:**
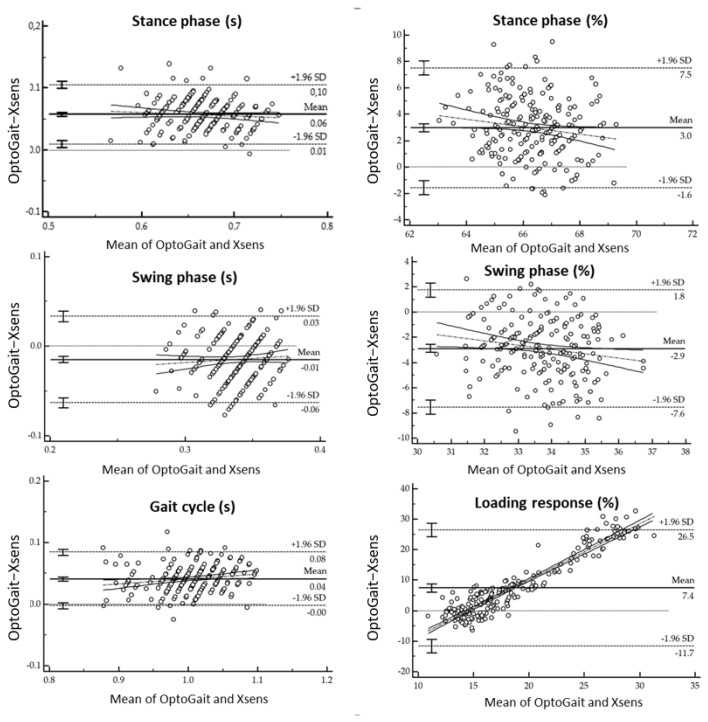
Bland-Altman plots for the difference gait assessment (OptoGait™ and Xsens) on the ordinates versus gait SPT parameters on abscissas (mean of both methods). The solid horizontal line indicates the bias while the dashed horizontal lines indicate the limit of agreements as 95% confidence interval of bias.

**Figure 5:**
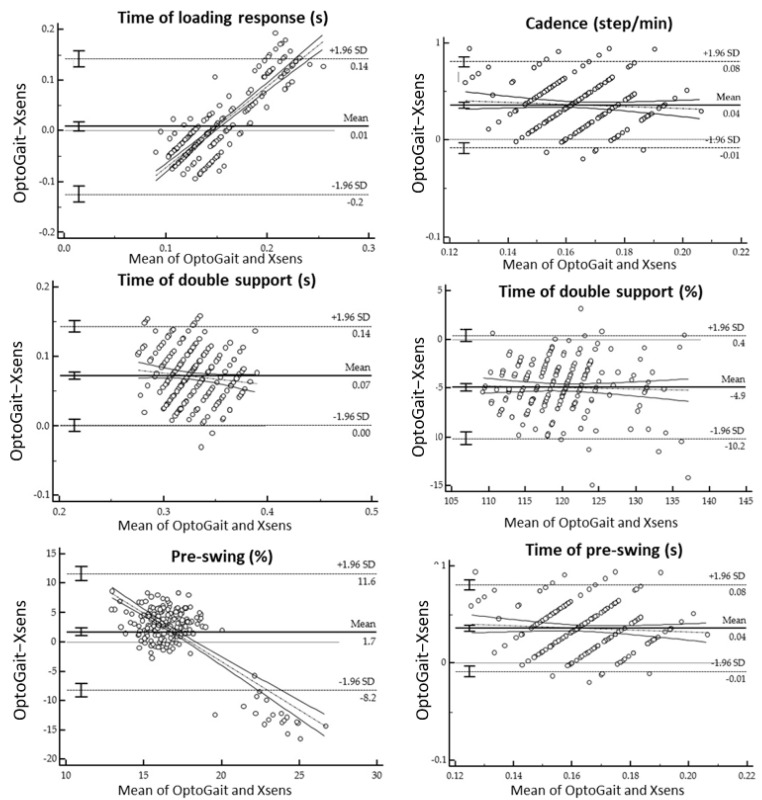
Bland-Altman plots for the difference gait assessment (OptoGait™ and Xsens) on the ordinates versus gait SPT parameters on abscissas (mean of both methods). The solid horizontal line indicates the bias while the dashed horizontal lines indicate the limit of agreements as 95% confidence interval of bias.

**Figure 6:**
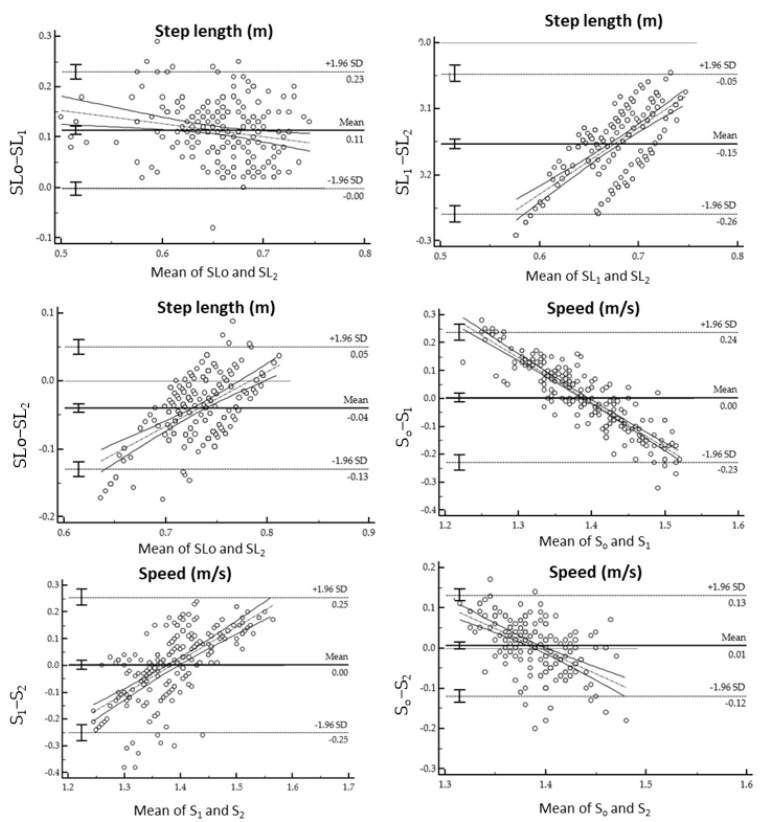
Bland-Altman plots for the difference gait assessment (OptoGait™ and Xsens) on the ordinates versus gait SPT parameters on abscissas (mean of both methods). The solid horizontal line indicates the bias while the dashed horizontal lines indicate the limit of agreements as 95% confidence interval of bias.

Spatiotemporal parameters measured with respectively the wearable inertial mocap system and the OptoGait^TM^ on the treadmill are shown in Table 2, Table 3, Table 4 and Table 5. Furthermore, Table 2 and Table 4 show the mean of differences, absolute agreement and consistency with the 95% confidence intervals and *p*-values. Meanwhile, Figure 4, Figure 5 and Figure 6 display the Bland-Altman plots analysis. Regression analysis of the difference together with the 95% confidence intervals (CI), *p*-values and coefficient of repeatability (CR) of the spatiotemporal parameters between Xsens and the OptoGait^TM^ are reported in Table 3 and Table 5.

Finally, Table 6 shows the mean square errors (MSE) considering S_1_ = speed calculated using the Equation (2), S_2_ = speed calculated with Equation (4) and S_O_ = speed calculated using the OptoGait^TM^ with respect to the imposed speed on treadmill (5 km/h).

## 4. Discussion

From this study emerged good agreement and consistency for the time of the stance phase and swing phase (Table 2). Although differences were found between the two systems as shown in Table 2 and Table 3. According to Lee et al. [32,54], a longer stance phase was found in the OptoGait^TM^ system. This can be caused as stated by previous studies by the fact that there is a gap between the treadmill belt and the Optogait^TM^ bars [32]. This causes an incorrect detection of the initial contact and toe-off [54]. This issue can affect the entire gait cycle and the relative phases. This can, in part, be solved by minimizing the space between treadmill belt and the bar integrating the bar in the treadmill. Nowadays, Microgait (Bolzano, Italy) offers the possibility of integrating the Optogait^TM^ bars on the treadmill as with Lode (Katana model, Lode BV, Netherlands.) or H/P Cosmos (quasar® model, Germany). However, this constitutes a non-portable solution that is costly. Regardless of this, Optogait^TM^ is affected, in addition, by the error due to an early heel contact and late toe-off caused by the LEDs that are raised by 3 mm from the floor [55,56]. However, a study from Lienhard et al. [27] also found this problematic issue in overground walking. In fact, according to Lienhard et al. [27], the error increases with decreasing walking speed. Despite this, different studies use this system as an optical system to assess agreement and reliability [27,32,54,55,56]. However, the two devices present a different sampling frequency that can have an effect on the precision of the event detection. According to Marmelat et al. [57], gait should be collected at 120 Hz for having a good compromise between accuracy and processing time. Nonetheless, this effect was not studied in this research testing different sampling frequencies due to the limitation of setting this technology at different sampling frequencies. Despite this, excellent agreement and consistency were found for the gait cycle and the cadence (Table 2). This was demonstrated by a gait cycle duration of 0.98 secs using the Xsens and the gait cycle duration of 1.02 s using the OptoGait^TM^. These results are in accordance with Murray et al. [58] (where the average gait cycle duration should correspond to values between 0.98 to 1.07 secs for men) and with the findings of Rudisch [29] (where high agreement and consistency were found). Poor agreement and consistency (Table 2 and Table 3) were found with the time of double support, time of loading and time of pre-swing between the two devices. In fact, systematic differences of these sub-phases (Table 2 and Table 3) can reflect a non-accurate acquisition of the heel-strike and toe-off event [29].

Regarding the step length and speed, different methods were presented. A method that calculates the step length using the inverted pendulum model applied on Xsens, SL_1_ (Equation (1)). In addition, a method that is based on the anthropometric approach (Equation (3)) that does not consider the step length variation. This equation is normally used in a pedometer but the application of this formula in gait analysis brings some limitations especially when we use the variation of the step length for evaluating pathologies [59]. Poor agreement but good consistency was found between SL_1_ (Xsens) and SLo (OptoGait^TM^). Differences between the two systems were also found with *p* < 0.0001 (Table 4). In term of step length, the OptoGait ^TM^ system does not consider the anthropometric variables, such as stature or leg length, but only the contact as interruption between the bars (Table 4). Step length calculated using the Xsens was underestimated with respect the OptoGait^TM^ and the anthropometric approach (Table 4). To investigate more, mean square errors (MSE) with respect to the imposed speed on treadmill of 5 km/h were also calculated for both devices measuring systems (Table 6). OptoGait^TM^ demonstrated being the device with lower S_O_ (MSE = 0.0008 m/s) with respect to the other devices. This is followed by the anthropometric approach S_2_ (MSE = 0.003 m/s) and the speed calculated using the Xsens, S_1_ (MSE = 0.014 m/s). Considering the two speeds using the Xsens and the anthropometric approach, two calibration factors as α and γ for each speed calculation S_1_ and S_2_ were adopted reducing this error. However, between the two systems, OptoGait^TM^ was revealed to be more accurate for step length and speed detection. 

In terms of capturing, the two devices request a different pre-acquisition period necessary for the setup and the calibration. Xsens requires the body placement of the wearable inertial sensors attached to the body by straps. This is after introducing the anthropometric measurements to the MVN Awinda software for representing and scaling the Digital Human Model (DHM) of the subject, where a successive calibration is required [60,61,62,63]. This is not practical when you need to acquire a patient in a clinical setting as you have limitation in time. However, the OptoGait^TM^ system does not requires such a long time since is not necessarily to place any sensor to the body. This is advantageous in terms of comfort because the subject does not wear any sensors that can influence their performance. In contrast, the OptoGait^TM^ does not permit a complete evaluation of the Kinematics of the subjects as the Xsens. The advantage of this device is the capability of exporting from this device information regarding 3D position, linear and angular acceleration and velocity of twenty-three segments: the 3D joint angles of twenty-two joints, the centre of mass of the body, the 3D orientation, free accelerations and magnetic data of the seventeen wearable inertial systems. In addition, Xsens can be used for monitoring different distances and terrains giving more complete freedom of capturing. This is not possible using the Optogait^TM^ as it is necessary to have the two receiving bars on the pathway. The bars need to be parallel and at the same distance, also creating a fixed pathway that may condition the protocol and the user’s performance. Furthermore, the Xsens offers the possibility of exporting the file as BVH files that can be imported into simulation software as Anybody for musculoskeletal simulation [63,64].

Additionally, this study presents some limitations. The first limitation is the use of only one imposed speed of 5 km/h for also evaluating the error between these devices. Future study needs to evaluate the error in speed by also considering different body placement of the sensor in Xsens using different imposed speeds. Secondly, the frequency of acquisition that maybe have been caused also differs in timing for the phases and sub-phases. The Xsens system does not offer the possibility to test the effect of sampling frequency in gait accuracy. This is because only two frequencies of acquisitions can be assessed as 60 Hz for full body and 100 Hz for the upper or lower body. A lower body configuration can present a benefit in term of accuracy in timing in phases and sub-phases. By contrast, this does not represent the full capturing for determining the full body kinematics and the exporting of it as BVH file necessary for the simulation of the musculoskeletal prediction in Anybody [64]. Third, in this study, we did not compare the two IGA instruments with a third IGA system as an optical system. This is because we saw that, in the literature, the OptoGait^TM^ system was used as a reference system to study agreement and reliability [27,32,54,55,56]. However, from this study, it emerged that OptoGait^TM^ also presents limitations due to the system design and integration. Overall, this study is important because it demonstrates the criticality of using these two different measurements systems in clinical trials.

## 5. Conclusions

In this paper, two different methods to measure spatiotemporal parameters using two systems were compared: a photoelectronic one (OptoGait^TM^) and a wearable inertial mocap system (Xsens). MSE calculation with respect to the imposed speed of 5 km/h shows a smaller error (MSE = 0.0008 m/s) using the OptoGait^TM^. Although this system proved to not be accurate on the calculation of the gait phases such as time and percentage of the stance phase and their sub-phases, agreement and consistency were found between the two systems for the calculation of the stance phase, cadence ad gait cycle. Nevertheless, an incorrect detection of toe-off and heel strike in OptoGait^TM^ caused by LEDs and the system design and integration and interaction with the treadmill can cause a systematic difference in phases and sub-phases. In addition, we proposed two methods: a method based on the inverted pendulum model for the calculation of gait phases and sub-phases applying it to Xsens and a method based on the anthropometric approach that does not consider the two instruments of measurements. In both cases for the speed, a calibration factor was determined for this specific speed. This calibration factor reduced the error in speed using the Xsens and the anthropometric approach. As the anthropometric approach is used in pedometers, we do not suggest using it when in the case it is necessary to evaluate the variation of the step length for clinical evaluation [59]. Moreover, OptoGait^TM^ presented a smaller MSE error for speed detection. 

Overall, our results indicate that the accurate detection of heel strike and toe-off have an influence on phases and sub-phases for the entire acquisition. Future study in this domain should investigate how to design and integrate better products and algorithms aiming to solve the problematic issues already identified in this study without limiting the user’s need and performance in a different environment. 

## Figures and Tables

**Table 1:** Anthropometric characteristics of the population (Mean, SD = standard deviation).

	Mean (SD)
Age (years)	25.42 (5.83)
Stature (m)	1.81 (0.06)
Body mass (kg)	74.92 (7.40)
BMI (kg/m^2^)	22.80 (1.36)
Leg length (m)	0.94 (0.05)

**Table 2:** Mean, SD (standard deviation), Mean of differences of the measured values between pairs of devices together with the 95% confidence intervals (CI) and *p*-values. Intraclass correlation coefficients (ICC) for mean of different gait variables measured for overlapping phases of system pairs. ICCs reflect absolute agreement (ICC_A) and consistency (ICC_C) of ratings.

SPT	Mean (SD) OptoGait^TM^	Mean (SD)Xsens	Mean Diff	95% CI	*p*	ICC_A Mean	95% CI	ICC_C Mean	95% CI
Stance phase (s)	0.69 (0.002)	0.63 (0.002)	0.056	0.053 to 0.0602	<0.0001	0.512	−0.153 to 0.819	0.87	0.829 to 0.901
Swing phase (s)	0.33 (0.001)	0.34 (0.001)	−0.014	−0.018 to −0.011	<0.0001	0.527	0.236 to 0.691	0.60	0.479 to 0.697
Stance phase (%)	67.80 (0.107)	64.83 (0.124)	2.971	2.655 to 3.287	<0.0001	0.026	−0.082 to 0.140	0.06	−0.224 to 0.289
Swing phase (%)	32.27 (0.104)	35.16 (0.124)	−2.888	−3.213 to −2.564	<0.0001	−0.028	−0.147 to 0.094	−0.074	−0.041 to 0.189
Gait cycle (s)	1.02 (0.003)	0.98 (0.003)	0.041	0.038 to 0.044	<0.0001	0.796	−0.167 to 0.940	0.945	0.928 to 0.958
Double support (%)	35.53 (0.171)	29.64 (0.188)	5.884	5.391 to 6.378	<0.0001	0.016	−0.061 to 0.101	0.056	−0.023 to 0.241
Time of Double support (s)	0.36 (0.002)	0.29 (0.002)	0.072	0.067 to 0.077	<0.0001	0.149	−0.123 to 0.403	0.461	0.293 to 0.590
Loading response (%)	22.27 (0.673)	14.85 (0.115)	7.423	6.093 to 8.752	<0.0001	0.028	−0.014 to 0.193	0.044	−0.254 to 0.272
Time of loading response (s)	0.15 (0.004)	0.14 (0.001)	0.008	−0.0005 to 0.017	0.0660	0.137	−0.129 to 0.341	0.138	−0.131 to 0.343
Pre−swing (%)	17.80 (0.114)	16.11 (0.321)	1.686	0.995 to 2.377	<0.0001	−0.122	−0.433 to 0.125	−0.137	−0.491 to 0.133
Time of pre−swing (s)	0.18 (0.001)	0.14 (0.001)	0.036	0.033 to 0.039	<0.0001	0.138	−0.118 to 0.362	0.362	0.162 to 0.514
Cadence (step/min)	117.50 (0.410)	122.39 (0.416)	−4.888	−5.256 to −4.520	<0.0001	0.804	−0.164 to 0.942	0.946	0.925 to 0.959

**Table 3:** Regression analysis of the differences (Coefficient (slope and intercept), SE = standard error) together with the 95 % confidence intervals (CI), *p*-values and coefficient of repeatability (CR) of the spatiotemporal parameters between the OptoGait^TM^ (O) and the Xsens (X).

SPT (O-X)	Parameter	Coefficient	SE	t	*p*	95% CI	CR	95% CI
Stance phase (s)	Intercept	0.096	0.033	2.900	0.0041	0.030 to 0.162	0.121	0.110 to 0.134
	Slope	−0.059	0.049	−1.192	0.2346	−0.157 to 0.038		
Swing phase (s)	Intercept	−0.046	0.029	−1.569	0.1182	−0.104 to 0.011	0.056	0.0514 to 0.0623
	Slope	0.093	0.087	1.065	0.2878	−0.079 to 0.265		
Stance phase (%)	Intercept	21.820	8.805	2.478	0.0140	4.461 to 39.180	7.378	6.733 to 8.160
	Slope	−0.284	0.132	−2.141	0.0334	−0.545 to −0.0225		
Swing phase (%)	Intercept	8.821	4.792	1.840	0.0671	−0.627 to 18.269	7.327	6.687 to 8.104
	Slope	−0.347	0.142	−2.444	0.0153	−0.627 to −0.0672		
Gait Cycle (s)	Intercept	−0.039	0.032	−1.219	0.2239	−0.102 to 0.0242	0.091	0.083 to 0.101
	Slope	0.080	0.032	2.496	0.0133	0.016 to 0.143		
Double support (s)	Intercept	0.127	0.033	3.826	0.0002	0.061 to 0.193	0.158	0.144 to 0.175
	Slope	−0.168	0.101	−1.666	0.0971	−0.368 to 0.030		
Double support (%)	Intercept	11.653	4.390	2.654	0.0086	2.997 to 20.308	13.531	12.349 to 14.966
	Slope	−0.177	0.134	−1.316	0.1896	−0.442 to 0.088		
Pre-swing (s)	Intercept	0.054	0.018	2.964	0.0034	0.018 to 0.089	0.0834	0.076 to 0.0927
	Slope	−0.108	0.110	−0.977	0.3295	−0.326 to 0.110		
Pre-swing (%)	Intercept	29.749	1.602	18.564	<0.0001	26.590 to 32.909	10.447	9.534 to 11.554
	Slope	−1.654	0.093	−17.682	<0.0001	−1.839 to −1.470		
Loading response (%)	Intercept	−26.826	0.864	−31.042	<0.0001	−28.529 to −25.122	23.984	21.889 to 26.527
	Slope	1.844	0.044	41.031	<0.0001	1.756 to 1.933		
Cadence (step/min)	Intercept	−2.893	3.864	−0.748	0.4549	−10.512 to 4.725	10.935	9.980 to 12.095
	Slope	−0.016	0.032	−0.516	0.6058	−0.080 to 0.046		

**Table 4:** Mean of differences of the measured values between pairs of devices together with the 95% confidence intervals (CI) and *p*-values. Intraclass correlation coefficients (ICC) for mean of different gait variables measured for overlapping phases of system pairs. ICCs reflect absolute agreement (ICC_A) and consistency (ICC_C) of ratings.

SPT	Mean A	Mean B	Mean Diff	95% CI	*p*	ICC_A Mean	95% CI	ICC_C Mean	95% CI
SLo (m), SL_1_ (m)	0.71 (0.50)	0.59 (0.721)	0.113	0.105 to 0.121	<0.0001	0.22	−0.156 to 0.534	0.579	0.447 to 0.679
SLo (m), SL_2_ (m)	0.71 (0.50)	0.75 (0.151)	−0.039	−0.046 to −0.033	<0.0001	0.366	−0.033 to 0.588	0.491	0.338 to 0.616
SL_1_ (m), SL_2_ (m)	0.59 (0.721)	0.75 (0.151)	−0.153	−0.160 to −0.145	<0.0001	0.088	−0.075 to 0.140	0.463	0.295 to 0.591
So (m/s), S_1_ (m/s)	1.39 (0.18)	1.38 (3.701)	0.003	−0.012 to 0.020	0.6421	0.171	−0.089 to 0.369	0.171	−0.088 to 0.368
So (m/s), S_2_ (m/s)	1.39 (0.18)	1.38 (0.631)	0.006	−0.002 to 0.014	0.1700	0.093	−0.434 to 0.167	−0.093	−0.435 to 0.167
S_1_ (m/s), S_2_ (m/s)	1.38 (3.701)	1.38 (0.631)	0.002	−0.015 to 0.019	0.8012	0.122	−0.153 to 0.332	0.122	−0.152 to 0.331

**Table 5:** Regression analysis of the difference (Coefficient (slope and intercept), SE = standard error) together with the 95% confidence intervals (CI), *p*-values and coefficient of repeatability (CR) of the spatiotemporal parameters between Xsens and the OptoGait^TM^.

	Parameter	Coefficient	SE	t	*p*	95% CI	CR	95% CI
SLo (m), SL_1_ (m)	Intercept	0.282	0.058	4.863	<0.0001	0.168 to 0.397	0.250	0.228 to 0.277
	Slope	−0.258	0.088	−2.921	0.0039	−0.433 to −0.084		
SLo (m), SL_2_ (m)	Intercept	−0.629	0.059	−10.577	<0.0001	−0.746 to −0.51	0.119	0.108 to 0.131
	Slope	0.805	0.081	9.915	< 0.0001	0.645 to 0.965		
SL_1_ (m), SL_2_ (m)	Intercept	−0.831	0.050	−16.598	<0.0001	−0.930 to −0.732	0.318	0.290 to 0.352
	Slope	1.004	0.074	13.558	<0.0001	0.858 to 1.150		
So (m/s), S_1_ (m/s)	Intercept	2.252	0.080	27.974	<0.0001	2.093 to 2.411	0.232	0.212 to 0.257
	Slope	−1.618	0.057	−27.957	<0.0001	−1.732 to −1.503		
S_1_ (m/s), S_2_ (m/s)	Intercept	−1.726	0.135	−12.702	<0.0001	−1.9943 to −1.458	0.252	0.230 to 0.278
	Slope	1.246	0.097	12.734	<0.0001	1.053 to 1.439		
So (m/s), S_2_(m/s)	Intercept	−0.629	0.059	−10.577	<0.0001	−0.746 to −0.512	0.119	0.108 to 0.131
	Slope	0.805	0.081	9.915	<0.0001	0.645 to 0.965		

**Table 6:** Mean square errors (MSE) considering S_1_ = speed calculated using the Equation (2). S_2_ = speed calculated with Equation (4) and S_O_ = speed calculated using the OptoGait^TM^ respect to the imposed speed on treadmill (5 km/h).

	S_1_	S_2_	S_O_
MSE (m/s)	0.014	0.003	0.0008

## Abbreviations

SPT	spatiotemporal parameters
IMU	inertial measurement unit
IGA	instrumented gait analysis
SD	standard deviation
COM	center of mass
DS	double support
Min	minimum
Max	maximum
ICC	intraclass correlation coefficients
ICC_A	intraclass correlation coefficients reflect absolute agreement
ICC_C	intraclass correlation coefficients reflect consistency
MAD	mean absolute deviation
MSE	mean square error
CI	confidence interval
